# Effectiveness of sensory integration-based intervention in autistic children, focusing on Chinese children: a systematic review and meta-analysis

**DOI:** 10.3389/fpsyt.2025.1623149

**Published:** 2025-11-19

**Authors:** Bingchen Lyu, Yi Ba, De Ma, Niu Liu, Limin Fu, Yaqi Xue

**Affiliations:** 1 Second Affiliated Middle School International Department, Beijing Normal University, Beijing, China; 2 College of Physical Education and Sports, Beijing Normal University, Beijing, China; 3 Department of Sport Arts, Hebei Sport University, Shijiazhuang, China

**Keywords:** autism spectrum disorder, sensory integration-based intervention, meta-analysis, children, systematic review

## Abstract

**Objective:**

In recent years, the prevalence of autism spectrum disorder (ASD) has been increasing year by year, bringing huge economic and mental burdens to society and families. Ayres Sensory Integration Intervention (ASI) is a widely used approach in the treatment of ASD. A more common sensory integration approach in China is called sensory integration-based intervention (SIBI), which is developed based on ASI. This study systematically analyzes the effects of SIBI on the sensory integration abilities and autism-related behaviors of autistic children.

**Methods:**

We searched PubMed, Cochrane Library, Web of Science, EBSCO, and CNKI databases from inception to February 2025. Randomized controlled trials (RCTs) using SIBI to improve autistic children were included. After selection, data were extracted on author, year, country, sample size, age, intervention type and duration, and outcome indicators. Quality assessment and data extraction were conducted independently by two researchers. Statistical analyses were performed in Stata 17, including forest plots, funnel plots with Egger’s test for publication bias, and meta-regression for heterogeneity.

**Results:**

A total of 16 studies with 1319 samples were included. The results showed that compared to the control group, SIBI had a positive effect on the total score of sensory integration ability scales for autistic children [mean difference (MD), 11.53; 95% confidence interval (CI) (10.53, 12.53); P<0.5]. Compared to the control group, SIBI also positively improved the total score of the ATEC scale [MD, -16.12; 95% CI (-22.61, -9.64); P<0.05] and the total score of the ABC scale [MD, -7.47; 95% CI (-11.69, -3.25); P<0.05]. The results from Egger’s test and the funnel plot indicated no publication bias. Due to the high heterogeneity (I²>50%) in the results of SIBI intervention on the total ABC score, a meta-regression analysis was conducted. The results of the meta-regression analysis indicated that age was not the source of heterogeneity, while the intervention duration was found to be the source of heterogeneity (β=-0.51, P<0.05, 95% CI [-1.01, -0.01]).

**Conclusion:**

SIBI can effectively improve the sensory integration ability and autism-related behaviors of autistic children.

**Systematic Review Registration:**

https://www.crd.york.ac.uk/PROSPERO/, identifier CRD420250639991.

## 1 Introduction

Autism spectrum disorder (ASD) is a common, highly heritable, and heterogeneous neurodevelopmental disorder that encompasses conditions such as autism, Asperger syndrome, and pervasive developmental disorder. These disorders are characterized by underlying cognitive features and frequently co-occur with other conditions (1). The core symptoms of ASD include social communication difficulties and repetitive patterns of behavior, interests, or activities. It is often accompanied by sensory integration disorders, emotional issues, and language communication difficulties (2). The etiology of ASD is complex and not yet fully understood, involving a combination of genetic and environmental factors (3). Early environmental insults may lead to abnormal brain structure and function, affecting neurodevelopmental processes and increasing the risk of ASD (4). At present, the prevalence of ASD is increasing annually, with the World Health Organization (WHO) estimating a global prevalence of 0.76% (5). In 2023, the Centers for Disease Control and Prevention (CDC) reported a prevalence rate of 1 in 36 among 8-year-old children in the United States (6). In China, epidemiological studies indicate that the prevalence of ASD among school-aged children (6–12 years) is 0.7%, suggesting that nearly 700, 000 children in China are affected by ASD (7). The increasing incidence of ASD, with its early and often lifelong onset, places an enormous economic and emotional burden on families and society. The “Analysis of the Current Situation of Autistic Children in China” report highlights that the monthly rehabilitation costs for children with ASD account for half of a family’s total expenditure. Currently, there are no specific pharmacological treatments for ASD, with medications such as risperidone and aripiprazole being used primarily for symptom management. As a result, ASD interventions mainly rely on non-pharmacological approaches, including Applied Behavior Analysis (ABA), the Early Start Denver Model, Ayres Sensory Integration^®^ (ASI) and so on, which form an essential component of the overall treatment strategy (8, 9).

Sensory integration is a process that involves combining sensory information received from various senses in the human body. After this information is processed and integrated into the central nervous system, it enables individuals to perceive their internal and external environments and triggers appropriate physical responses. However, individuals with sensory integration dysfunction may make errors in processing sensory stimuli accurately. While sensory factors are not specific elements for diagnosing autism, they can affect the vast majority of autistic children (10, 11). Sensory integration dysfunction mainly includes tactile defensive disorder, motor difficulties, auditory language disorders, vestibular balance dysfunction, and spatial and structural perception disorders (12). The sensory system issues in autistic children are significant factors affecting other behavioral problems, such as social skills, social engagement, and academic performance (13). Therefore, improving the sensory system function in autistic children is also a crucial measure to enhance the overall treatment effectiveness.

ASI, developed by A. Jean Ayres, is a therapeutic approach designed to enhance sensory integration functions by influencing the way the brain processes and organizes sensory information through the provision of controlled sensory stimuli in the context of playful and meaningful activities (14). ASI is a clinical-based approach that focuses on the relationship between the therapist and the child, using play-based sensory-motor activities aimed at improving sensory processing and integration (15). Currently, ASI has been widely applied in research involving children with various diseases, with ASD being one of them (16–18). ASI can effectively improve the social skills and self-care abilities of autistic children (19, 20).At the same time, occupational therapy using the principles of ASI is one of the most requested services by parents of autistic children (21). A survey of occupational therapists also showed that 82% of respondents indicated they ‘always’ use sensory integration methods in their treatment when working with autistic children (22). Several systematic reviews have examined the impact of ASI on autistic children, focusing on issues such as the fidelity of intervention implementation, the strength and certainty of the available evidence, and the reproducibility of intervention procedures (23–25). In China, a more commonly used sensory integration approach is referred to as sensory integration-based intervention (SIBI), which was developed based on the ASI framework. However, despite its widespread application, research conducted in China remains limited in both quality and consistency, characterized by relatively small sample sizes and variability in outcome measurement tools. Therefore, our review aims to contribute by providing quantitative evidence specific to Chinese populations, thereby complementing the broader international literature.

Therefore, to address this potential issue, this meta-analysis conducted searches in both Chinese and English databases to supplement the lack of sufficient RCTs, with the hope of providing a theoretical basis for the effects of SIBI interventions on autistic children. This study aims to synthesize published research to analyze the impacts of SIBI on the sensory integration abilities and behaviors of autistic children, exploring the specific effects of SIBI on this population.

## 2 Methods

Meta-analysis is performed according to the preferred reporting items in the System Review and Meta-analysis (PRISMA) guide (26).(CRD420250639991).

### 2.1 Literature search strategy

The studies search was performed for the related research studies, mainly from the following databases: PubMed, Cochrane Library, Web of Science, EBSCO, CNKI. The search keywords we used were (“ayres sensory integration intervention OR sensory integration therapy OR ayres sensory integration OR sensory integration training” OR SIT OR SII OR AIT OR “sensory integration”) AND (autism OR ASD OR autism spectrum disorder) AND (RCT OR random OR random controlled trial). This is limited to February 2025.

### 2.2 Inclusion and exclusion criteria

The following PICOS criteria were employed for the selection of studies included in the research.

Participant (p): Inclusion criteria for research subjects are children under the age of 18 diagnosed with ASD in hospitals. Also does not include other mental illnesses.
Intervention (I): using SIBI therapy to intervene in ASD.
Comparison (C): In the control group, autistic children only received standard care or did not receive any intervention.
Outcomes (O): outcomes indicators containing ABC (Autism Behavior Checklist)OR ATEC (Autism Treatment Evaluation Checklist) OR SIS(Sensory Integration Scale).
Study design (S): Randomized Controlled Trials, Controlled Studies, Studies with.

Criteria for exclusion: (1) observational designs rather than randomized controlled trials; (2) involved non-human subjects include rats, mice, etc.; (3) included children with ADHD or other neurodevelopmental disorders instead of ASD; (4) The outcome measures do not include ATEC, ABC, SIS; and (5) inability to extract outcome indicators.

### 2.3 Data extraction

Two authors independently screened the abstracts and full-text articles of the selected works, extracted and cross-verified the data. In instances of disagreement, a third researcher was consulted to mediate and reach consensus. During the studies screening process, the initial step involved reading the titles and abstracts, followed by a thorough examination of the full texts to determine the studies to be excluded.

### 2.4 Quality assessment

Two researchers independently screened literature, extracted relevant data, and conducted cross checks. Disagreements were resolved through discussion or, if necessary, with the assistance of a third researcher. The extracted data includes basic research information, characteristics of research participants, specific details of interventions, measured outcome indicators, and research quality. Evaluating the methodological quality of included studies using the Physical Therapy Evidence Database (PEDro) scale(https://pedro.org.au/simplified-chinese/resources/pedro-scale/)There are a total of 11 evaluation items. Meeting each standard earns 1 point and does not meet or apply any standards earns 0 points. Research with a score of 0–3 is classified as low-quality, 4–7 as moderate quality, and 8–11 as high-quality (27).

### 2.5 SIBI description

The SIBI currently used and most widely practiced in China are culturally adapted programs derived from Ayres’ sensory integration approach (14, 28). These interventions were originally developed in 1992 through a collaboration between the Institute of Mental Health at Peking Medical University and Professor Wen-De Chen, who analyzed and modified Ayres’ model to create an integrated program suited to the Chinese context (28).

### 2.6 Statistical analysis

We used Stata 17.0 to conduct a meta-analysis on the data from all included studies. All outcome indicators selected in this study were continuous variables, and there were no differences among the outcome measurement tools used in individual studies. Therefore, the weighted mean difference (MD) was used for calculation. The criteria for judging heterogeneity were as follows: When I² ≤ 50%, it indicates that the meta-analysis results show low heterogeneity, and a fixed-effect model should be applied. When I² > 50%, it indicates that the meta-analysis results have high heterogeneity, and a random-effect model should be used (29). Additionally, we conducted a meta-regression analysis on results with high heterogeneity to explore the sources of that heterogeneity. Subsequently, a funnel plot was constructed, and Egger’s test was used to analyze publication bias. If publication bias was detected, the trim and fill method was used to correct it.

## 3 Result

### 3.1 Search results

**Figure 1:**
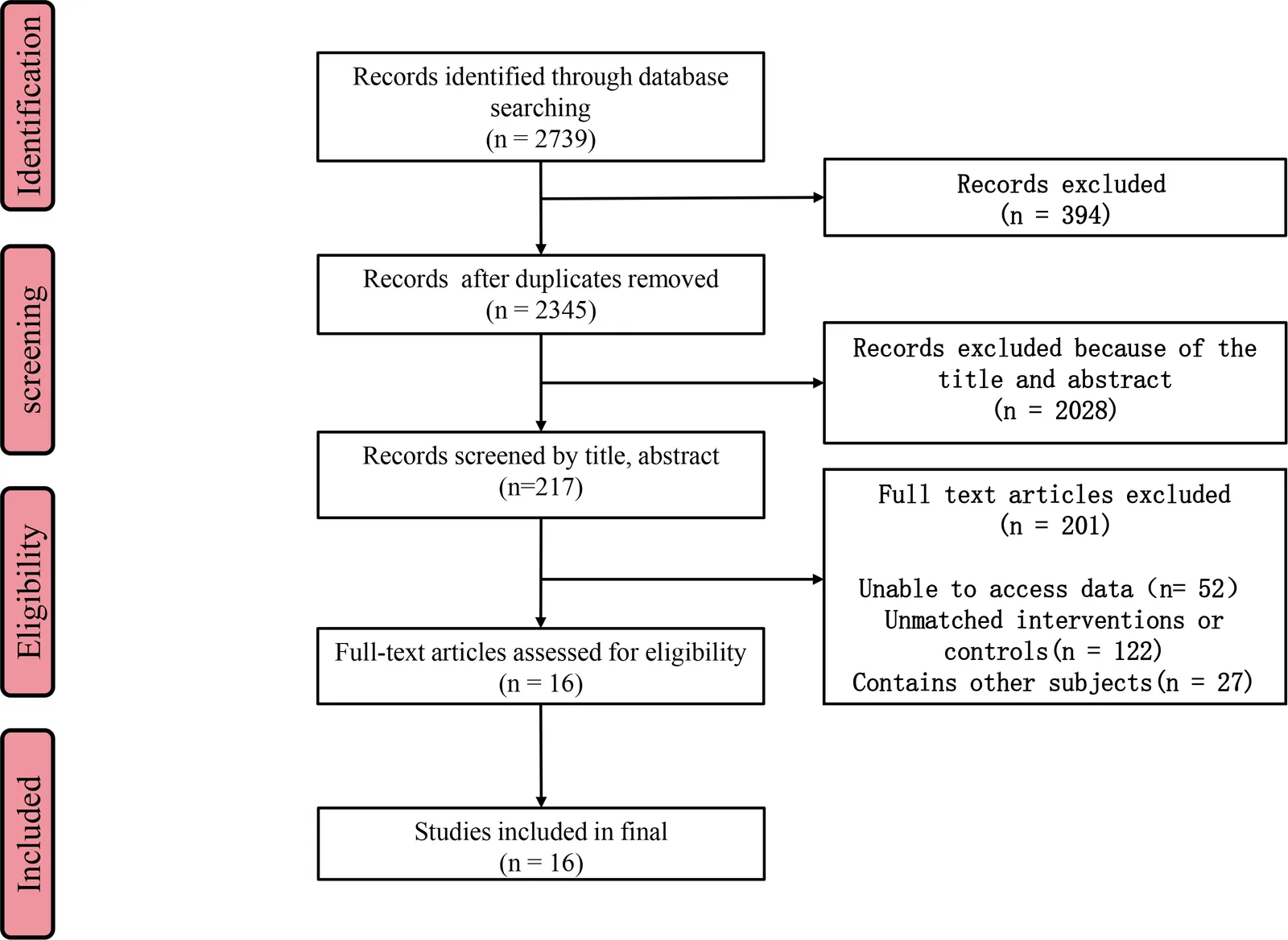
Flow chart of literature search and screening.

A total of 2739 studies were initially collected from the database for meta-analysis. After removing 394 duplicate studies, 2345 studies remained. Following the evaluation of titles and abstracts, 2028 studies were excluded. Subsequently, a full-text review of 217 potentially relevant studies was conducted, during which 201 studies were deemed ineligible. Ultimately, only 16 publications met the inclusion criteria. **Figure 1** provides the steps involved in the screening procedure.

### 3.2 Study characteristics

A total of 16 studies included 1, 319 ASD patients, with 645 in the control group and 674 in the intervention group. All ASD patients were diagnosed in hospitals or through specialized diagnostic scales, with an average age of under 10 years. The duration of SIBI ranged from 12 to 24 weeks, with the frequency of intervention sessions varying from 3 to 7 times per week, and each session lasting between 40 and 240 minutes. Five studies used the ATEC as an outcome measure, eleven studies used the ABC as an outcome measure, and six studies used the SIS as an outcome measure (**Table 1**). The PEDro results indicate that the quality scores of all literature ranged from 5 to 7, suggesting medium-quality literature (**Supplementary Table 1**). The specific components of the SIBI applied in the included studies are presented in **Supplementary Table 2**.

**Table 1:** Characteristics of the included studies.

Author, year	SI assessment	Participants	Region	Age	N	Intervention	Cycle	Outcomes	PEDro
				C/T	C/T	C/T			
Deng Hongzhu, 2003 (30)	NO	DSM-IV diagnosis	China	6.48 ± 2.87/ 7.11 ± 1.96	15/30	N/SIBI	90min, 4t, 24w	ATEC	6
Yang Hong, 2009 (31)	NO	DSM-IV diagnosis	China	6.24 ± 2.62/ 7.21 ± 1.86	14/28	N/SIBI	40min, 7t, 24w	ATEC	6
Chen Lingjiao, 2017 (32)	YES	DSM-IVdiagnosis	China	6.9 ± 1.1	34/34	N/SIBI	240min, 7t, 24w	ABC, SIS	7
Liu Long, 2017 (33)	NO	Hospitaldiagnosis	China	7.38 ± 5.14/8.52 ± 5.49	30/30	NT/SIBI +NT	90min, 4t, 24w	ATEC	6
Jin Xin, 2018 (34)	YES	Hospitaldiagnosis	China	03-Dec	40/40	NT/SIBI+NT	240min, 7t, 24w	ATEC, SIS	7
Li Xiaoyan, 2018 (35)	NO	Hospitaldiagnosis	China	7.56 ± 2.1/ 7.16 ± 2.37	20/20	N/SIBI	Total120t, 24w	ATEC	5
Zhang Yanmin, 2018 (36)	NO	Hospitaldiagnosis	China	8.3 ± 1.0/ 8.5 ± 1.1	20/20	NT/SIBI +NT	40min, 7t, 24w	ABC	6
He Fengying, 2019 (37)	YES	Hospitaldiagnosis	China	5.9 ± 2.6	46/46	NT/SIBI +NT	240min, 7t, 24w	ABC, SIS	6
Huang Yimin, 2019 (38)	YES	DSM-IVdiagnosis	China	5.8 ± 1.4/6.2 ± 1.3	75/75	NT/SIBI +NT	Total84t, 12w	ABC, SIS	5
Li Haiyu, 2019 (39)	YES	DSM-IVdiagnosis	China	5.1 ± 1.5/5.4 ± 1.4	79/79	NT/SIBI +NT	120min, 6t, 12w	ABC, SIS	6
Li Huihui, 2021 (40)	YES	DSM-IVdiagnosis	China	8.3 ± 0.2/ 8.4 ± 0.3	41/41	NT/SIBI +NT	40min, 60t, 24w	ABC, SIS	6
Wan Kai, 2021 (41)	NO	Hospitaldiagnosis	China	8.06 ± 0.67/7.94 ± 0.53	58/58	NT/SIBI +NT	75min, 3t, 12w	ABC	6
Zhang Xiaoyu, 2022 (42)	NO	DSM-Vdiagnosis	China	3.71 ± 0.61/3.8 ± 0.65	60/60	NT/SIBI +NT	120min, 7t, 12w	ABC	6
Zhang Guixin, 2020 (43)	NO	Hospitaldiagnosis	China	8.62 ± 1.54/8.16 ± 1.29	31/34	NT/SIBI +NT	60min, 3t, 24w	ABC	7
Pi Xiang, 2020 (44)	NO	DSM-Vdiagnosis	China	5.11 ± 1.01/5.08± 1.02	29/29	NT/SIBI +NT	7t, 24w	ABC	5
Wenxin Xu, 2019 (45)	NO	CCMD-3diagnosis	China	6.18 ± 2.94/6.17 ± 2.44	53/50	NT/SIBI +NT	7t, 12w	ABC	6

NT, normal treatment; N, none; C, control group; T, treatment group; t, times; w, week.

### 3.3 The effect of SIBI on SIS in autistic children

**Figure 2:**
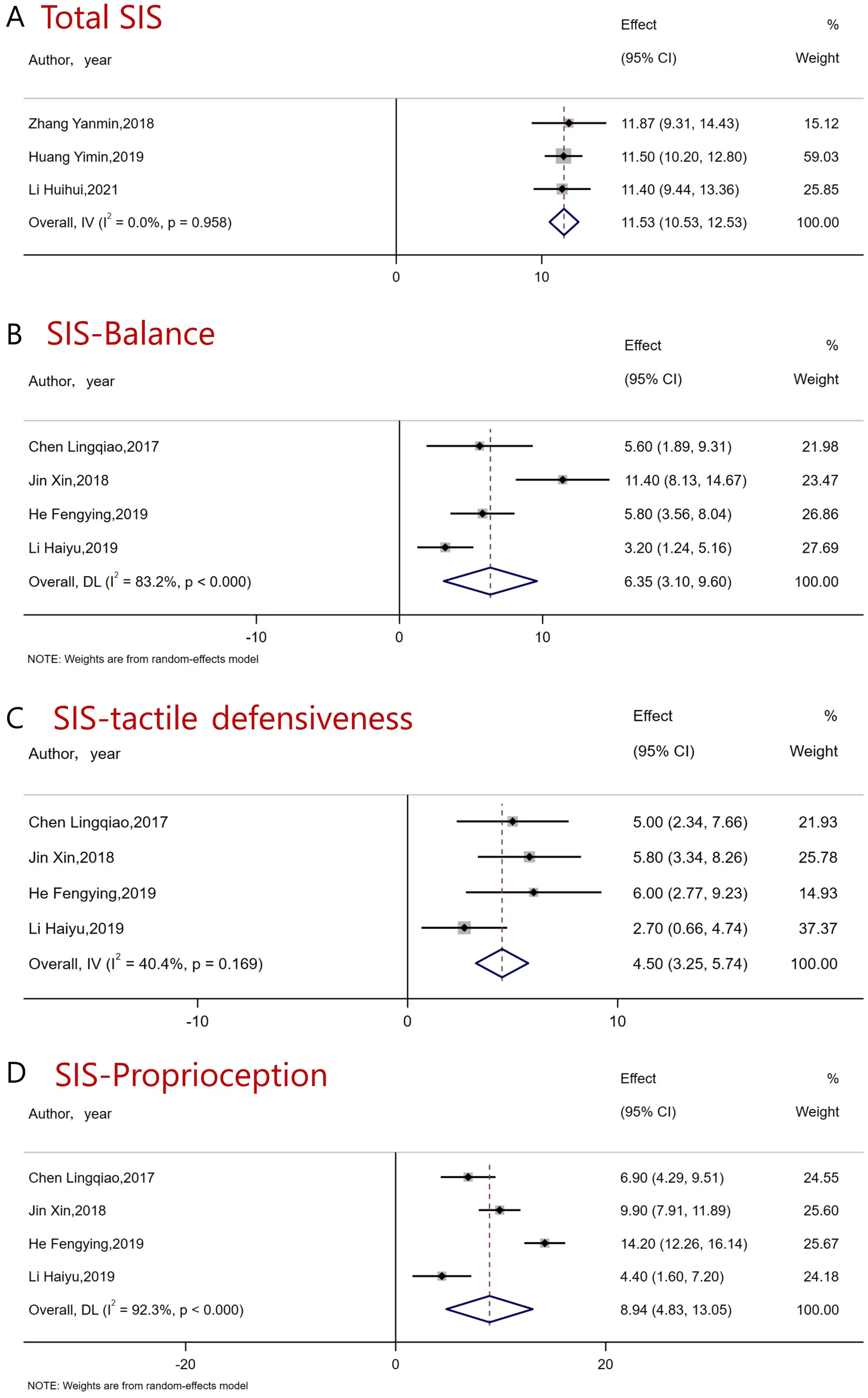
Forest plot of the effects of SIBI intervention on sensory integration abilities in autistic children. **(A)** Total SIS **(B)** Balance **(C)** Tactile defensiveness **(D)** Proprioception.

The SIS is used to assess children’s sensory integration abilities and includes three dimensions, vestibular balance, tactile defensiveness, and proprioception. SIS was used as an outcome measure to evaluate the effect of SIBI on sensory integration ability in autistic children. The SIS was developed by Professor Zheng Xinxiong from Taiwan, based on the checklist presented in “Sensory Integration and the Child” from the University of Southern California (46). The SIS has since been widely applied in clinical and research settings across China. In terms of total SIS scores, 3 studies met the inclusion criteria, with 136 participants receiving SIBI and 136 participants assigned to the control group. (**Figure 2A**). Due to statistically insignificant heterogeneity (I^2^ = 0%, P>0.05), a fixed-effect model was applied in the meta-analysis. Considering the combined effects, a statistically significant improvement was observed [MD = 11.53, 95% CI (10.53, 12.53)]. A diamond-shaped square on the right side of the zero line crosses it, indicating that compared to the control group, the SIBI effectively enhances sensory integration ability in children with ASD.

First, in the balance dimension, a total of 4 studies met the inclusion criteria and were included. Due to statistically significant heterogeneity (I^2^ = 83.2%, P<0.05), a random-effect model was applied in the meta-analysis. The results showed that balance ability in autistic children was significantly improved after SIBI [MD = 6.35, 95% CI (3.10, 9.60)] (**Figure 2B**). Then, in the dimension of tactile defensiveness, a total of four studies met the inclusion criteria and were included. Due to statistically insignificant heterogeneity (I^2^ = 40.4%, P>0.05), a fixed-effect model was applied in the meta-analysis. The results showed that tactile defensiveness in autistic children was significantly improved after SIBI [MD = 5.50, 95% CI (3.25, 5.47)] (**Figure 2C**). Finally, in the proprioception dimension, a total of four studies met the inclusion criteria and were included. Due to statistically significant heterogeneity (I^2^ = 92.3%, P<0.05), a random-effects model was applied in the meta-analysis. The results showed that proprioception in autistic children significantly improved after SIBI [MD = 8.94, 95% CI (4.83, 13.50)] (**Figure 2D**). Overall, although high heterogeneity was observed in the balance and proprioception subdimensions, the results for the total SIS score were robust with low heterogeneity. This indicates that SIBI can significantly enhance the sensory integration abilities of autistic children.

### 3.4 The effect of SIBI on ATEC in autistic children

**Figure 3:**
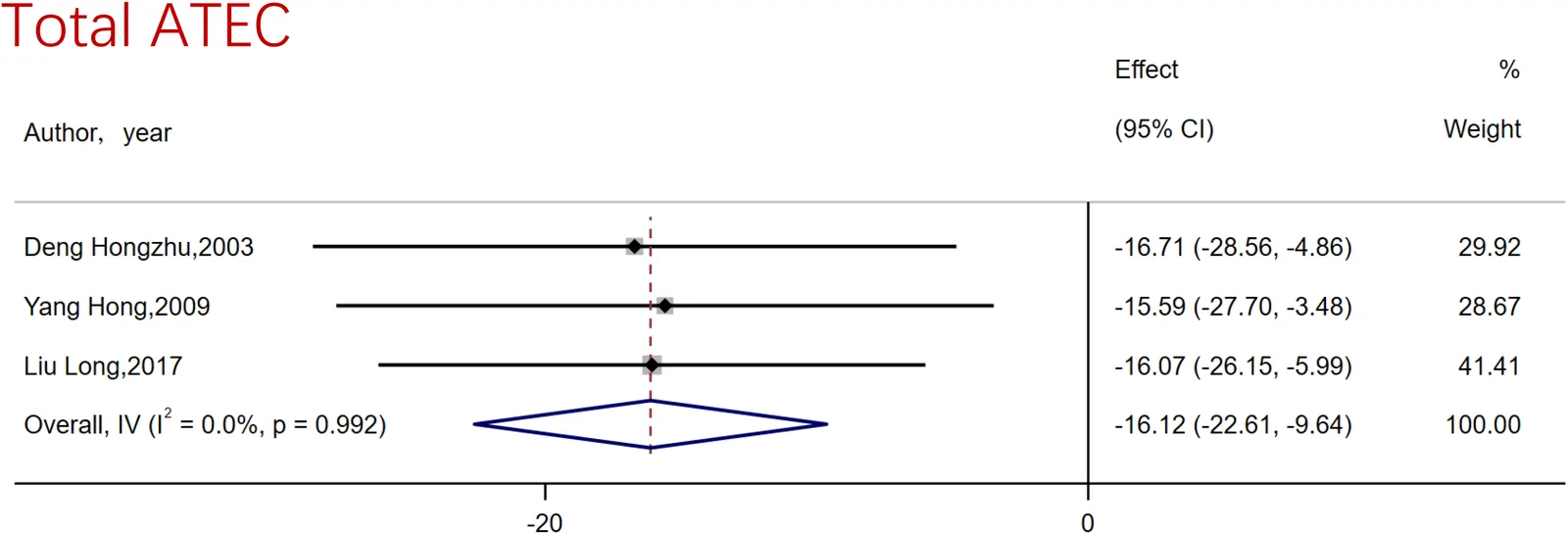
Forest plot of the effects of SIBI on ATEC in autistic children.

The ATEC assessment scale is a tool for evaluating symptoms and treatment effects in autistic children. It covers the performance of autistic children in perception/cognitive ability, social ability, behavior, and communication aspects. Regarding the total ATEC score, 3 studies met the inclusion criteria, with 90 participants receiving SIBI intervention and 59 participants assigned to the control group. (**Figure 3**). On account of statistically insignificant heterogeneity (I^2^ = 0%, P>0.05), a fixed-effect model was used for meta-analysis. The results showed that SIBI effectively reduced the total ATEC score of autistic children [MD=-16.12, 95%CI (-22.61, -9.64)], indicating that SIBI has a significant alleviating effect on autism-related behaviors in autistic children. Firstly, in perception/cognitive ability, due to statistically insignificant heterogeneity (I^2^ = 0%, P>0.05), a fixed-effect model was used for meta-analysis. The results showed that SIBI effectively improved the perception/cognitive ability score of autistic children [MD=-3.81, 95%CI (-5.22, -2.40)]. Secondly, in social ability, 4 studies met the inclusion criteria. Due to statistically insignificant heterogeneity (I2 = 0%, P>0.05), a fixed-effect model was used for meta-analysis. The results showed that SIBI could effectively improve the social ability of autistic children [MD=-1.81, 95%CI (-3.07, -0.55)]. Thirdly, regarding behavior, 4 studies met the inclusion criteria. Due to statistically insignificant heterogeneity (I^2^ = 0%, P>0.05), a fixed-effect model was used for meta-analysis. The results showed that SIBI could effectively improve the behavioral abilities of autistic children [MD=-3.99, 95%CI (-5.51, -2.47)]. Finally, in terms of communication skills, 4 studies met the inclusion criteria. Due to statistically insignificant heterogeneity (I^2^ = 0%, P>0.05), a fixed-effect model was used for meta-analysis. The results showed that SIBI could effectively improve the communication skills of autistic children [MD=-2.62, 95%CI (-3.87, -1.36)] (**Table 2**). Overall, SIBI has a significant effect on improving the total scores of ATEC and the scores of various sub-dimensions in autistic children, with low heterogeneity and robust results. This indicates that SIBI can significantly improve the autistic behaviors of autistic children.

**Table 2:** The impact of SIBI on various sub-dimensions of ATEC in autistic children.

ATEC subgroup	n	I^2^	Model	Results	
				SMD [95%CI]	*P*
Perception/cognitive ability	4	0	Fixed model	-3.81 [-5.22, -2.40]	*P* < 0.05
Social ability	4	40.2	Fixed model	-1.81 [-3.07, -0.55]	*P* < 0.05
Behavior	4	48	Fixed model	-3.99[-5.51, -2.47]	*P* < 0.05
Communication	4	31.6	Fixed model	-2.62[-3.87, -1.36]	*P* < 0.05

### 3.5 The effect of SIBI on ABC in autistic children

**Figure 4:**
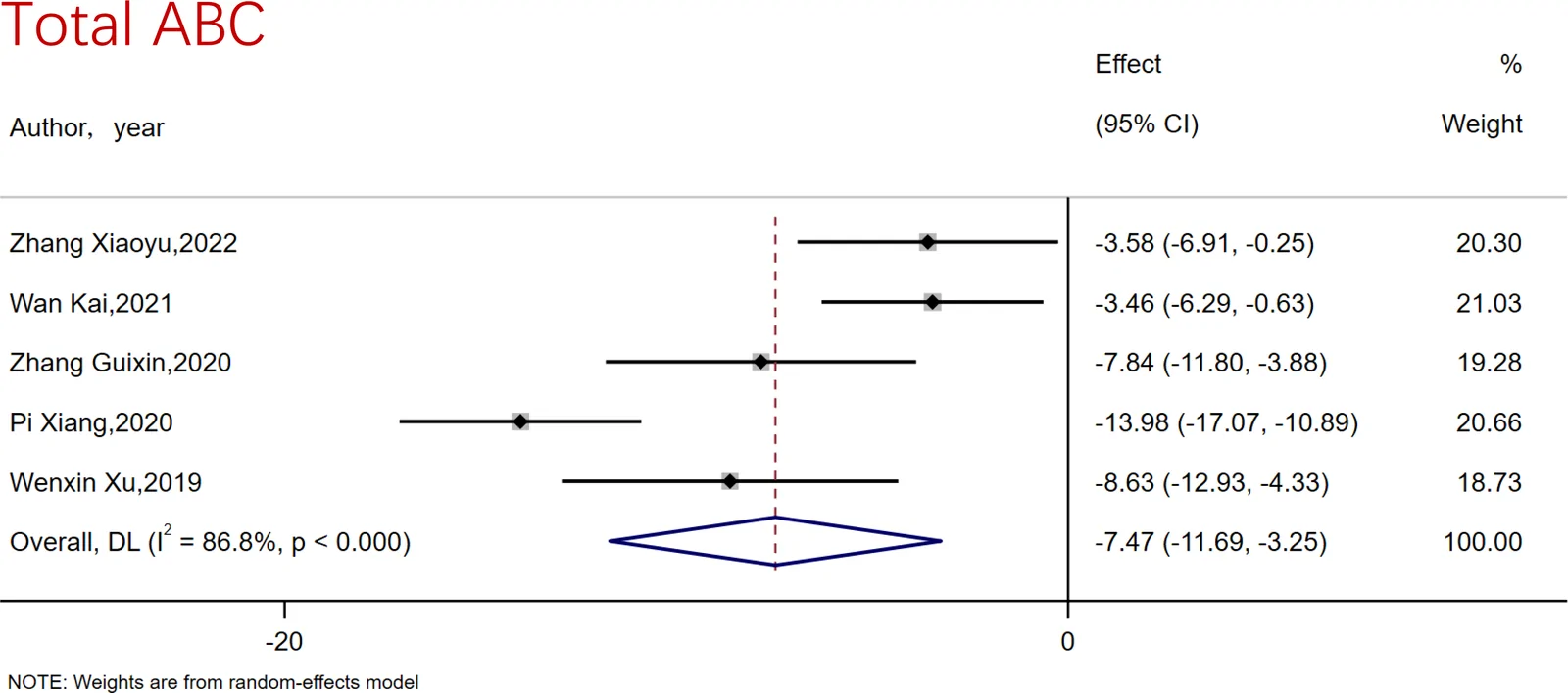
Forest plot of the effects of SIBI on ABC in autistic children.

The ABC assessment scale serves as a tool to evaluate the severity of autistic children, encompassing sensory, physical movement, social skills, self-care, and language abilities. Concerning the total ABC score, 5 studies met the inclusion criteria, with 231 participants receiving SIBI and 231 participants assigned to the control group. The result of heterogeneity analysis indicated significant heterogeneity (I^2^ = 86.8%) (**Figure 4**). A random-effects model was employed for meta-analysis. The findings reveal that SIBI effectively reduces the total ABC score in autistic children [MD=-7.47, 95%CI (-11.69, -3.25)], suggesting that SIBI beneficially modifies autistic behaviors in these children (**Figure 4**). Firstly, in the area of Sensory, a total of 7 studies met the inclusion criteria. The heterogeneity results indicated statistically significant heterogeneity (I^2^ = 72.5%, P<0.05), and a random-effects model was employed for the meta-analysis. The results showed that SIBI can effectively reduce sensory issues in autistic children [MD=-1.58, 95%CI (-2.05, -1.11)]. Secondly, regarding Somatic movement, 7 studies met the inclusion criteria. The results indicated insignificant heterogeneity (I^2^ = 22.4%, P>0.05), and a fixed-effects model was used for statistical analysis. The results demonstrated that SIBI can significantly improve Somatic movement issues in autistic children [MD=-1.37, 95%CI (-1.67, -1.06)]. Thirdly, in the domain of social skills, 7 studies met the inclusion criteria. The results revealed significant heterogeneity (I^2^ = 74.6%, P>0.05), and a random-effects model was applied for statistical analysis. The findings showed that SIBI can significantly alleviate social skills issues in autistic children [MD=-2.28, 95%CI (-3.08, -1.49)]. Then, in terms of Self-care, 6 studies met the inclusion criteria. The results indicated significant heterogeneity (I^2^ = 90.5%, P<0.05), and a random-effects model was utilized for statistical analysis. The results demonstrated that SIBI can significantly enhance Self-care issues in autistic children [MD=-2.42, 95%CI (-3.99, -0.84)]. Finally, in the area of language, 5 studies met the inclusion criteria. The results showed significant heterogeneity (I^2^ = 83.6%, P<0.05), and a random-effects model was employed for statistical analysis. The findings indicated that SIBI can significantly improve language issues in autistic children [MD=-1.53, 95%CI (-1.86, -1.19)] (**Table 3**). Overall, SIBI has a significant effect on improving the total scores of ABC and the scores of various sub-dimensions in autistic children, but there is high heterogeneity, and the results are not robust, with only the Somatic movement sub-dimension showing low heterogeneity.

**Table 3:** The impact of SIBI on various sub-dimensions of ABC in autistic children.

ABC subgroup	n	I^2^	Model	Results		
				SMD [95%CI]	*P*
Sensory	7	72.5	Random model	-1.58 [-2.05, -1.11]	*P* < 0.05
Somatic movement	7	22.4	Fixed model	-1.37[-1.67, -1.06]	*P* < 0.05
Social skills	7	74.6	Random model	-2.28[-3.08, -1.49]	*P* < 0.05
Self-care	6	90.5	Random model	-2.42[-3.99, -0.84]	*P* < 0.05
Language	5	83.6	Random model	-1.53[-1.86, -1.19]	*P* < 0.05

### 3.6 Publication bias detection

**Figure 5:**
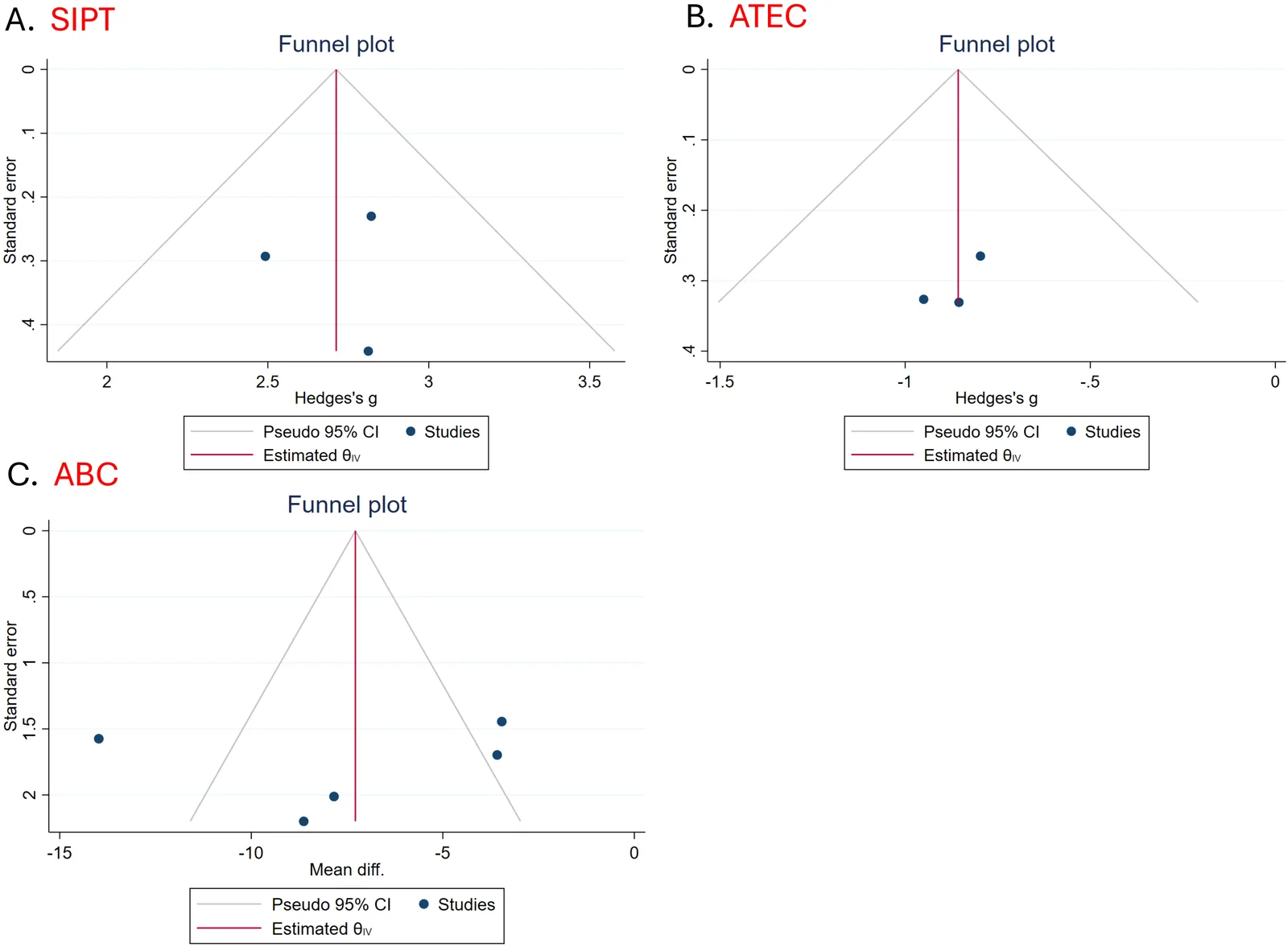
Funnel plot **(A)** Funnel plot of the impact of SIBI on SIS **(B)** Funnel plot of the impact of SIBI on ATEC **(C)** Funnel plot of the impact of SIBI on ABC.

In this study, the Egger test and funnel plots were utilized to assess publication bias. Regarding the improvement of SIS by SIBI, the Egger test results indicated p>0.05, suggesting no evidence of publication bias. In the context of SIBI’s effect on improving the total ATEC score, the Egger test results also showed p>0.05, indicating no publication bias. Similarly, for the improvement of the total ABC score by SIBI, the Egger test results revealed p>0.05, signifying no publication bias. Concurrently, the analysis of the funnel plot yielded consistent results, as illustrated in **Figure 5**.

### 3.7 Meta regression analyses

**Figure 6:**
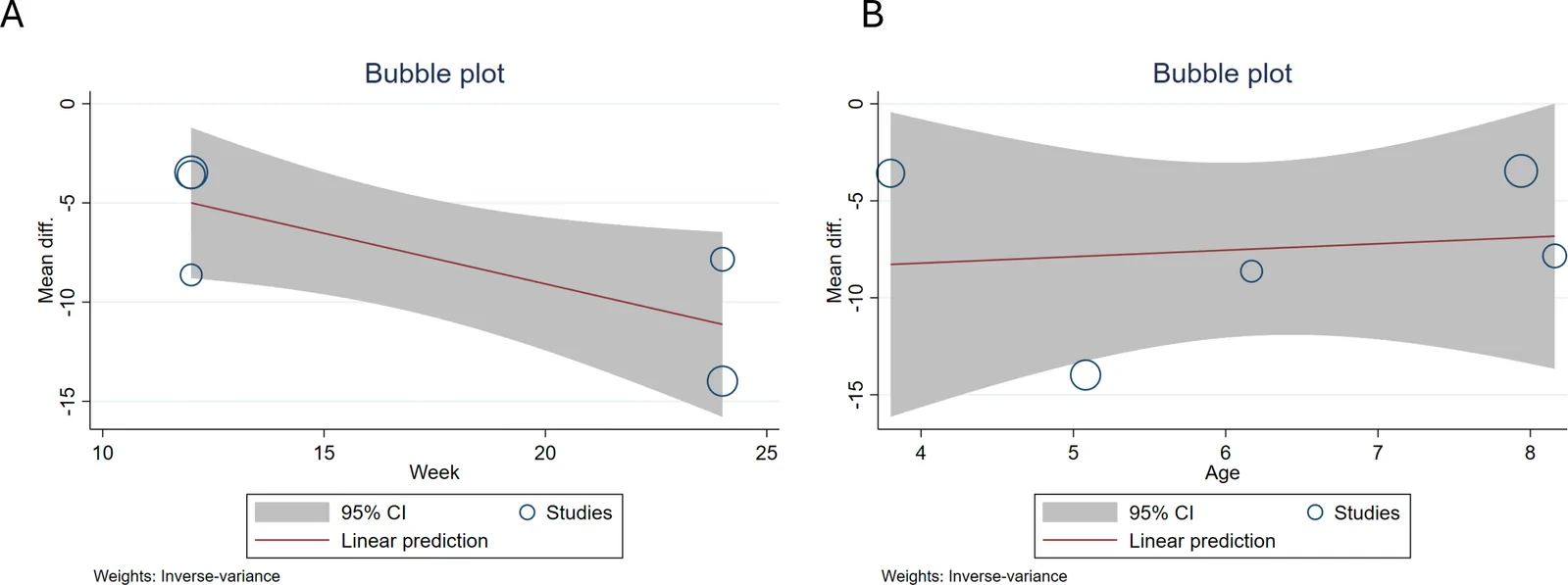
Meta-regression analysis bubble plot of the effects of SIBI on ABC in autistic children **(A)** The impact of the number of intervention weeks on the results. **(B)** The impact of age on the results.

In order to examine the heterogeneity of the effects of SIBI on the total ABC score in autistic children, a meta-regression analysis was conducted to identify variables that may influence the intervention outcomes. The results of the regression analysis indicated that age did not have a significant effect on the total score of the ABC scale improved by SIBI in autistic children (β=0.33, *P*>0.05, 95%CI[-2.33, 3.00]) (**Figure 6B**). However, the number of intervention weeks had a significant impact on the effectiveness of SIBI in reducing the total ABC score, demonstrating that a longer duration of intervention weeks was associated with a greater reduction in the total ABC score (β=-0.51, *P*<0.05, 95%CI[-1.01, -0.01]) (**Figure 6A**).

## 4 Discussion

This meta-analysis investigated the impact of SIBI on sensory processing abilities and autism-related behaviors in autistic children. The analysis provides empirical evidence regarding the efficacy of SIBI in enhancing sensory processing capabilities and ameliorating autism-related behaviors.

Our research findings indicate that SIBI can effectively enhance the sensory integration abilities of autistic children in three aspects: balance, tactile defensiveness, and proprioception. Additionally, the heterogeneity test results show that the improvement of the total SIS score for autistic children through SIBI is robust (I^2^<50%). In normally developing children, sensory integration dysfunction significantly impacts learning, behavior, and social communication. This phenomenon can be ameliorated through targeted interventions (47). SIBI is a method that helps individuals regulate their neurological functions and promotes the integration and processing of environmental information through systematic intervention. This approach is based on the principles of neuroplasticity, facilitating the brain’s hierarchical processing of multisensory signals by integrating information received from different sensory modalities (19). As a result, it coordinates the body’s adaptive responses to external environmental stimuli and internal physiological states. By reinforcing the information integration capabilities of the nervous system through targeted training, SIBI can effectively enhance children’s behavioral organization levels, specifically reflected in improvements in joint attention, increased proactivity in social interactions, enhanced motor coordination, and improved sensory discrimination skills (48). The SIS scale encompasses sensory integration ability assessments across three dimensions: balance, tactile defensiveness, and proprioception. Maintaining balance necessitates the integration of diverse information from the vestibular, visual, and proprioceptive systems to effectively sustain equilibrium (49). Individuals with autism face greater challenges in balance (50). Further research indicates that postural and balance difficulties are related to other autistic characteristics, such as social communication deficiencies and repetitive behaviors (51, 52). SIBI appears to have a compensatory effect on sensory input deficits in children with ASD, particularly in the areas of visual, vestibular perception, and proprioception. SIBI can enhance the balance abilities of children with ASD by modulating the corresponding posture control mechanisms in different brain regions (25). Moreover, fNIRS evidence indicates that the enhanced neural activation in specific regions of the PFC highlights the role of SIBI in cognitive improvement, including inhibitory control and cognitive flexibility (53). However, this emerging compensatory effect deserves further exploration in future studies utilizing more sophisticated neuroscientific instruments.

Sensory integration disorder is not exclusive to individuals with ASD, but it is crucial to study SIBI in ASD children because this anomaly is commonly found in ASD patients and has a relatively high prevalence. Some atypical sensory behaviors can be clearly observed in autistic children in the early stages of life (around 1 year old), and these behaviors are more common in infancy and childhood than in adulthood (54). Research has shown that children with ASD exhibit several different atypical sensory patterns, including hyperresponsiveness (HYPER), hyporesponsiveness (HYPO), and sensory interests, repetitions, and seeking behaviors (SIRS), as well as the potential role of sensory processing as a crucial component of higher social and cognitive functions. Additionally, the atypical sensory behaviors displayed by individuals with sensory integration disorder are also part of the diagnostic criteria for core symptoms of autism spectrum disorder in DSM-5. Individuals with ASD exhibit abnormalities in central sensory processing, and these atypical characteristics related to the integration of somatosensory information are associated with an increase in autistic traits, further supported by the neurobiological characteristics of ASD (55, 56). The sensory processing abnormalities in children with ASD not only impair their sensory integration abilities but also have a significant impact on their behavioral issues, including core symptoms (12). Therefore, improving the sensory integration abilities of individuals with ASD may have significant benefits for the improvement of their core symptoms.

SIBI is a program that can enhance their sensory integration abilities. In individuals with autism, SIBI can reduce stereotypical behaviors, improve self-care and social interaction abilities, and positively influence motor skills, socialization, attention, behavior control, reading skills, participation in play activities, and the achievement of personal goals (16, 20). The meta-analysis results also found similar outcomes. SIBI not only improves sensory integration abilities in autistic children but also alleviates their autistic behaviors. SIBI can effectively reduce the total scores of the ATEC scale and the ABC scale in autistic children. The ATEC scale includes four dimensions: perception/cognitive ability, social ability, behavior, and communication. The results show that SIBI can effectively reduce both the total score and the symptom scores of each sub-dimension on the ATEC scale, and importantly, the results are robust (I2 < 50%). Additionally, the ABC scale, which is an autistic behavior assessment scale that can be used by parents, includes 5 dimensions: Sensory, Somatic Movement, Social Skills, Self-Care, and Language. The meta-analysis also found that the SIBI can reduce the total score of the ABC scale and the scores of each sub-dimension for autistic children. However, the results of the meta-analysis showed high heterogeneity. We then used meta-regression to examine the sources of heterogeneity, and the results indicated that the intervention cycle of SIBI is a source of heterogeneity affecting the overall score of the ABC scale. At the same time, this result highlights that a longer intervention cycle is associated with better improvement effects on the total score of the ABC. These results indicate that SIBI can not only directly improve the sensory integration ability of children with autism but also enhance their social cognitive function by reinforcing these fundamental abilities, thereby improving their social skills, language skills, and self-care abilities. The potential mechanism underlying these results may be due to the continuous SIBI providing comfortable sensory stimulation for children with autism, enabling them to reach an appropriate level of arousal through sensory input, and that SIBI stimulation can develop their visual processing abilities, influencing changes in their psychological and social functions through changes in physical abilities (57, 58).

These findings underscore the importance of SIBI in enhancing sensory integration processing abilities and improving autism-related behaviors, providing support for non-pharmacological interventions in autism treatment based on neuroplasticity. However, there are still some difficulties in the implementation of SIBI for ASD currently, such as the distinction between SIBI and sensory-based interventions in practical applications. SIBI is an important rehabilitation approach for ASD and must be strictly performed according to ASI principles to ensure the effectiveness of the intervention.

Recent international systematic reviews and empirical studies on ASI have provided increasing evidence that this intervention can improve social participation, communication, and sensory processing in autistic children across different cultural contexts (23). Our findings based on Chinese studies are generally consistent with these outcomes, particularly in demonstrating improvements in core autistic behaviors and sensory integration function. However, several differences are noteworthy. First, the version of SIBI applied in China is an adaptation developed by Professor Wen-De Chen in collaboration with Peking Medical University, based on ASI and modified to fit the Chinese cultural context. Next, compared with international studies, Chinese trials often focus on symptom reduction scales such as the ABC or SIS rather than on functional outcomes. In contrast, contemporary international research has increasingly emphasized ecologically valid measures, including Goal Attainment Scaling (GAS), activities of daily living (ADLs), social skills, and reductions in caregiver burden. These similarities and differences suggest that while ASI holds promise in the Chinese context, future research would benefit from adopting internationally recognized fidelity measures and incorporating more functional outcome indicators. Such efforts would not only enhance the comparability of Chinese studies with global evidence but also provide stronger guidance for clinical practice.

Current guidelines for ASI research emphasize that assessment of ASI concerns should be conducted prior to intervention to ensure appropriate participant inclusion and accurate evaluation of treatment effects. However, in this review, we found that several included studies did not perform formal SI assessments before intervention, and some provided insufficient information regarding assessment procedures. This inconsistency reflects the fact that a number of studies were conducted before such standards were widely adopted. The absence of standardized pre-intervention SI assessment represents a major limitation of the present review and highlights the need for future studies to implement rigorous assessment protocols to enhance the validity of intervention outcomes. Although praxis difficulties are known to occur in a large proportion of children with autism and represent a core aspect of sensory integration, most of the studies included in this review did not specifically assess or report praxis-related outcomes. Consequently, the absence of praxis assessment limits the ability to fully evaluate the intervention’s effectiveness, and future studies should incorporate standardized measures of praxis to provide a more comprehensive understanding of sensory integration-based interventions in children with autism.

## 5 Conclusion

This study aims to provide theoretical evidence for the effectiveness of SIBI by exploring its impact on children with autism. The results indicate that SIBI can effectively improve the sensory integration ability and autism-related behaviors of autistic children. Furthermore, the intervention effects gradually increase with the duration of SIBI. Additionally, this study has some limitations, including that the sample comprised only Chinese children with autism, the number of studies included is relatively small, and dissertations were excluded. Future research needs to include larger samples and high-quality RCT experiments from an international perspective to support these findings.

## 6 Limitation

All randomized controlled trials were conducted in China. Therefore, these findings may have limited generalizability to other regions and cultural backgrounds.

## Data Availability

The original contributions presented in the study are included in the article/**Supplementary Material**. Further inquiries can be directed to the corresponding authors.
