# Correction: Effectiveness of sensory integration-based intervention in autistic children, focusing on Chinese children: a systematic review and meta-analysis

**DOI:** 10.3389/fpsyt.2026.1997362

**Published:** 2026-09-28

**Authors:** Bingchen Lyu, Yi Ba, De Ma, Niu Liu, Limin Fu, Yaqi Xue

**Affiliations:** 1 Second Affiliated Middle School International Department, Beijing Normal University, Beijing, China; 2 College of Physical Education and Sports, Beijing Normal University, Beijing, China; 3 Department of Sport Arts, Hebei Sport University, Shijiazhuang, China

**Keywords:** autism spectrum disorder, sensory integration-based intervention, meta-analysis, children, systematic review

In the published article, the pooled effect estimate for the total Autism Behavior Checklist (ABC) score was reported incorrectly in two places. The values from the Autism Treatment Evaluation Checklist (ATEC) analysis were inadvertently repeated for the ABC analysis. The incorrect estimate was “MD = -16.12, 95% CI (-22.61, -9.64).” The correct estimate for the total ABC score is “MD = -7.47, 95% CI (-11.69, -3.25).”

A correction has been made to the **Abstract**, *Results*. The original text reads:

“Compared to the control group, SIBI also positively improved the total score of the ATEC scale [MD, -16.12; 95% CI (-22.61, -9.64); P<0.05] and the total score of the ABC scale [MD, -16.12; 95% CI (-22.61, -9.64); P<0.05].”

The corrected text reads:

“Compared to the control group, SIBI also positively improved the total score of the ATEC scale [MD, -16.12; 95% CI (-22.61, -9.64); P<0.05] and the total score of the ABC scale [MD, -7.47; 95% CI (-11.69, -3.25); P<0.05].”

A correction has also been made to **3 Results**, *3.5 The effect of SIBI on ABC in autistic children*, paragraph 2. The original text reads:

“The findings reveal that SIBI effectively reduces the total ABC score in autistic children [MD=-16.12, 95%CI (-22.61, -9.64)], suggesting that SIBI beneficially modifies autistic behaviors in these children (**Figure 4**).”

The corrected text reads:

“The findings reveal that SIBI effectively reduces the total ABC score in autistic children [MD=-7.47, 95%CI (-11.69, -3.25)], suggesting that SIBI beneficially modifies autistic behaviors in these children (**Figure 4**).”

The original version of this article has been updated.

