# Supplementary material for: Effectiveness of sensory integration-based intervention in autistic children, focusing on Chinese children: a systematic review and meta-analysis
Source: Front Psychiatry. 2025 Nov 19;16:1623149. doi: 10.3389/fpsyt.2025.1623149 (PMC12673401; doi:10.3389/fpsyt.2025.1623149)
Supplement: Supplementary file 1 [file Table1.docx]

| Author，year | Participants | Region | Age | N | Intervention | 周期 | Outcomes |
| --- | --- | --- | --- | --- | --- | --- | --- |
|  |  |  | C/T | C/T | C/T |  |  |
| Deng Hongzhu,2003^[1]^ | DSM-IV diagnosis | China | 6.48±2.87/ 7.11±1.96 | 15/30 | N/SIT | 90min,4t,24w | ATEC |
| Yang Hong,2009^[2]^ | DSM-IV diagnosis | China | 6.24±  2.62/ 7.21±1.86 | 14/28 | N/SIT | 40min,7t,24w | ATEC |
| Chen Lingqiao,2017^[3]^ | DSM-IVdiagnosis | China | 6.9±1.1 | 34/34 | N/SIT | 240min,7t,24w | ABC,SIPT |
| Liu Long,2017^[4]^ | Hospitaldiagnosis | China | 7.38±5.14/8.52±5.49 | 30/30 | NT/SIT+NT | 90min,4t,24w | ATEC |
| Jin Xin,2018^[5]^ | Hospitaldiagnosis | China | 3-12 | 40/40 | NT/SIT+NT | 240min,7t,24w | ATEC，SIPT |
| Li Xiaoyan,2018^[6]^ | Hospitaldiagnosis | China | 7.56±2.1/ 7.16±2.37 | 20/20 | N/SIT | Total120t,24w | ATEC |
| Zhang Yanmin,2018^[7]^ | Hospitaldiagnosis | China | 8.3±1.0/ 8.5±1.1 | 20/20 | NT/SIT+NT | 40min,7t,24w | ABC |
| He Fengying,2019^[8]^ | Hospitaldiagnosis | China | 5.9±2.6 | 46/46 | NT/SIT+NT | 240min,7t,24w | ABC, SIPT |
| Huang Yimin,2019^[9]^ | DSM-IVdiagnosis | China | 5.8±1.4/6.2±1.3 | 75/75 | NT/SIT+NT | Total84t,12w | ABC, SIPT |
| Li Haiyu,2019^[10]^ | DSM-IVdiagnosis | China | 5.1±1.5/5.4±1.4 | 79/79 | NT/SIT+NT | 120min,6t,12w | ABC, SIPT |
| Li Huihui,2021^[11]^ | DSM-IVdiagnosis | China | 8.3±0.2/ 8.4±0.3 | 41/41 | NT/SIT+NT | 40min,60t,24w | ABC, SIPT |
| Wan Kai,2021^[12]^ | Hospitaldiagnosis | China | 8.06±0.67/7.94±0.53 | 58/58 | NT/SIT+NT | 75min,3t | ABC |
| Zhang Xiaoyu,2022^[13]^ | DSM-Vdiagnosis | China | 3.71±0.61/3.8±0.65 | 60/60 | NT/SIT+NT | 120min,7t,12w | ABC |
| Zhang Guixin,2020^[14]^ | Hospitaldiagnosis | China | 8.62±1.54/8.16±1.29 | 31/34 | NT/SIT+NT | 60min,3t,24w | ABC |
| Pi Xiang,2020^[15]^ | DSM-Vdiagnosis | China | 5.11±1.01/5.08± 1.02 | 29/29 | NT/SIT+NT | 7t,24w | ABC |
| Wenxin Xu,2019^[16]^ | CCMD-3diagnosis | China | 6.18±2.94/6.17±2.44 | 53/50 | NT/SIT+NT | 12w | ABC |

[1] 邓红珠, 邹小兵, 唐春*, et al.* 感觉统合训练治疗儿童孤独症的近期疗效研究 %J 实用医学杂志 %J THE JOURNAL OF PRACTICAL MEDICINE, 2003, **19**(9): 984-986.

[2] 杨宏. 感觉统合训练治疗儿童孤独征疗效影响因素分析 感觉统合训练治疗儿童孤独征疗效影响因素分析 2009, **33**(1): 35-37.

[3] 陈玲娇, 胡文辉, 陈棉. 感觉统合训练对孤独症患儿的感觉失调和行为症状的改善作用 %J 中国乡村医药 %J Chinese Journal of Rural Medicine and Pharmacy, 2017, **24**(22): 37-38.

[4] 刘龙. 感觉统合训练治疗儿童孤独症的疗效分析 %J 中国医药指南 %J Guide of China Medicine, 2017, **15**(10): 186.

[5] 靳鑫. 感觉统合训练对孤独症患儿的感觉失调和行为症状的改善作用 %J 饮食保健 %J Diet Health, 2018, **5**(14): 65-66.

[6] 李晓岩. 感觉统合训练治疗儿童孤独症疗效分析 %J 中医药临床杂志 %J Clinical Journal of Traditional Chinese Medicine, 2018, **30**(11): 2117-2119.

[7] 张艳敏, 尚清. 感觉统合训练在自闭症患儿康复中的应用效果观察 %J 中国民康医学 %J Medical Journal of Chinese People's Health, 2018, **30**(23): 78,83.

[8] 何风英, 刘笑婴, 卢晓岩*, et al.* 感觉统合训练对孤独症患儿的感觉失调和行为症状的改善作用 %J 特别健康 %J Special Health, 2019, 16): 219-220.

[9] 黄艺敏. 感觉统合训练对自闭症患儿康复效果的影响 %J 实用中西医结合临床 %J Practical Clinical Journal of Integrated Traditional Chinese and Western Medicine, 2019, **19**(10): 107-108.

[10] 李海玉. 感觉统合训练在自闭症患儿中的应用效果分析 %J 实用中西医结合临床 %J Practical Clinical Journal of Integrated Traditional Chinese and Western Medicine, 2019, **19**(12): 103-104.

[11] 李慧卉. 感觉统合训练对自闭症患儿的影响分析 %J 中国继续医学教育 %J China Continuing Medical Education, 2021, **13**(17): 93-96.

[12] 万凯, 尚清, 李靖婕*, et al.* 感觉统合训练对孤独症患儿平衡能力和ABC、CARS评分的影响 %J 中国实用医刊 %J Chinese Journal of Practical Medicine, 2021, **48**(20): 53-56.

[13] 张晓宇, 张艳蕾. 基于丹佛模式下的感觉统合训练对2～5岁孤独症谱系障碍患儿交往能力及父母育儿压力的影响 %J 中国中西医结合儿科学 %J Chinese Pediatrics Of Integrated Traditional And Western Medicine, 2022, **14**(1): 41-44.

[14] 张桂欣, 王耀. 感觉统合训练在高功能孤独症谱系障碍患儿治疗中的应用 %J 河南医学研究 %J Henan Medical Research, 2020, **29**(28): 5269-5271.

[15] 皮翔. 感觉统合训练在自闭症儿童认知能力及动作发展能力中的应用效果 %J 中国当代医药 %J China Modern Medicine, 2020, **27**(12): 103-106.

[16] Xu W, Yao J, Liu W. Intervention Effect of Sensory Integration Training on the Behaviors and Quality of Life of Children with Autism. Psychiatria Danubina, 2019, **31**(3): 340-346.
