# Supplementary material for: Effectiveness of sensory integration-based intervention in autistic children, focusing on Chinese children: a systematic review and meta-analysis
Source: Front Psychiatry. 2025 Nov 19;16:1623149. doi: 10.3389/fpsyt.2025.1623149 (PMC12673401; doi:10.3389/fpsyt.2025.1623149)
Supplement: Supplementary file 2 [file Table2.docx]

Table 2 Specific process of SII

| Author,  year | A problem with sensory integration | SII process | 10 key components of ASI |
| --- | --- | --- | --- |
| Deng Hongzhu,  2003 | **Not reported** | **Tactile function training:** Large-area skin stimulation was applied to children with autism using tactile balls, ball pools, and similar activities, with the aim of improving tactile function.  **Vestibular function training:** Children were guided to perform rotational exercises. In a standing position, they were instructed to rotate the whole body or specific body parts around a relatively fixed axis to the left or right while keeping the feet stationary. Additional activities included standing on suspended equipment such as swinging bridges or barrels to practice rotation, rolling on the ground in a supine position with simultaneous foot movements, trampoline jumping, prone vibration on a large therapy ball, and bouncing on a therapy ball to provide up-and-down and vibratory stimulation.  **Proprioceptive function training:** Initially, children were instructed to repeat single-movement exercises, such as prone sliding on a scooter board or back rolling on a large therapy ball. Subsequently, they practiced multi-movement sequences, followed by integrative activities such as rolling while pushing a ball or rolling while striking a ball.  **Social and language function training:** Training sessions incorporated playful scenarios to enhance interest and engagement. During the sessions, children engaged in simple dialogues, and their verbal expressions were corrected as needed. Positive reinforcement and encouragement were provided to children who demonstrated good performance.  **Lack of learning ability training.** | 1. Ensures physical safety.  **YES**  2. Presents sensory opportunities. **YES**  3. Helps the child to attain and maintain appropriate levels of alertness. **YES**  4. Challenges postural, ocular, oral, or bilateral motor control. **YES**  5. Challenges in praxis and organization of behavior **NO**  6. Collaborates in activity choice **NOT Clear**  7. Tailors activity to present just-right challenge  8. Ensures that activities are successful  9. Supports child’s intrinsic motivation to play. **YES**  10. Establishes a therapeutic alliance |
| Yang Hong,  2009 | **Not reported** | **Tactile function training:** Large-area skin stimulation was applied to children with autism using tactile balls, ball pools, and similar activities, with the aim of improving tactile function.  **Vestibular function training:** Children were guided to perform rotational exercises. In a standing position, they were instructed to rotate the whole body or specific body parts around a relatively fixed axis to the left or right while keeping the feet stationary. Additional activities included standing on suspended equipment such as swinging bridges or barrels to practice rotation, rolling on the ground in a supine position with simultaneous foot movements, trampoline jumping, prone vibration on a large therapy ball, and bouncing on a therapy ball to provide up-and-down and vibratory stimulation.  **Proprioceptive function training:** Initially, children were instructed to repeat single-movement exercises, such as prone sliding on a scooter board or back rolling on a large therapy ball. Subsequently, they practiced multi-movement sequences, followed by integrative activities such as rolling while pushing a ball or rolling while striking a ball.  **Lack of learning ability training.** | 1. Ensures physical safety. **YES**  2. Presents sensory opportunities. **YES**  3. Helps the child to attain and maintain appropriate levels of alertness. **YES**  4. Challenges postural, ocular, oral, or bilateral motor control. **YES**  5. Challenges in praxis and organization of behavior **NO**  6. Collaborates in activity choice **NOT Clear**  7. Tailors activity to present just-right challenge **YES**  8. Ensures that activities are successful **YES**  9. Supports child’s intrinsic motivation to play. **YES**  10. Establishes a therapeutic alliance **YES** |
| Chen LingJiao,2017 | **Yes** | Activities such as balance beam walking, sack jumping, trampoline exercises, sliding, and ladder climbing were employed to improve body coordination.  Activities included puzzles, object assembly, bead threading, and needle threading games.  **Visual, auditory, memory, and attention training:** Cards with words, figures, and numbers were used to attract children’s attention, prompting them to describe card contents, practice sequencing, and perform group-based tasks. Each session lasted 60 minutes and was conducted twice daily.  **Social and language function training:** Training sessions incorporated playful scenarios to enhance interest and engagement. During the sessions, children engaged in simple dialogues, and their verbal expressions were corrected as needed. Positive reinforcement and encouragement were provided to children who demonstrated good performance. | 1. Ensures physical safety. **YES**  2. Presents sensory opportunities. **YES**  3. Helps the child to attain and maintain appropriate levels of alertness. **YES**  4. Challenges postural, ocular, oral, or bilateral motor control. **YES**  5. Challenges in praxis and organization of behavior **Not clear**  6. Collaborates in activity choice **Not clear**  7. Tailors activity to present just-right challenge **YES**  8. Ensures that activities are successful **YES**  9. Supports child’s intrinsic motivation to play. **YES**  10. Establishes a therapeutic alliance **YES** |
| Liu Long,2017 | **Not reported** | **Tactile function training:** Large-area skin stimulation was applied to children with autism using tactile balls, ball pools, and similar activities, with the aim of improving tactile function.  **Vestibular function training:** Children were guided to perform rotational exercises. In a standing position, they were instructed to rotate the whole body or specific body parts around a relatively fixed axis to the left or right while keeping the feet stationary. Additional activities included standing on suspended equipment such as swinging bridges or barrels to practice rotation, rolling on the ground in a supine position with simultaneous foot movements, trampoline jumping, prone vibration on a large therapy ball, and bouncing on a therapy ball to provide up-and-down and vibratory stimulation.  **Proprioceptive function training:** Initially, children were instructed to repeat single-movement exercises, such as prone sliding on a scooter board or back rolling on a large therapy ball. Subsequently, they practiced multi-movement sequences, followed by integrative activities such as rolling while pushing a ball or rolling while striking a ball.  **Social and language function training:** Training sessions incorporated playful scenarios to enhance interest and engagement. During the sessions, children engaged in simple dialogues, and their verbal expressions were corrected as needed. Positive reinforcement and encouragement were provided to children who demonstrated good performance. | 1. Ensures physical safety. **YES**  2. Presents sensory opportunities. **YES**  3. Helps the child to attain and maintain appropriate levels of alertness. **YES**  4. Challenges postural, ocular, oral, or bilateral motor control. **YES**  5. Challenges in praxis and organization of behavior **NO**  6. Collaborates in activity choice **NOT Clear**  7. Tailors activity to present just-right challenge **YES**  8. Ensures that activities are successful **YES**  9. Supports child’s intrinsic motivation to play. **YES**  10. Establishes a therapeutic alliance **YES** |
| Jin Xin,2018 | **Yes** | **Tactile function training:** Large-area skin stimulation was applied to children with autism using tactile balls, ball pools, and similar activities, with the aim of improving tactile function.  **Vestibular function training:** Children were guided to perform rotational exercises. In a standing position, they were instructed to rotate the whole body or specific body parts around a relatively fixed axis to the left or right while keeping the feet stationary. Additional activities included standing on suspended equipment such as swinging bridges or barrels to practice rotation, rolling on the ground in a supine position with simultaneous foot movements, trampoline jumping, prone vibration on a large therapy ball, and bouncing on a therapy ball to provide up-and-down and vibratory stimulation.  **Proprioceptive function training:** Initially, children were instructed to repeat single-movement exercises, such as prone sliding on a scooter board or back rolling on a large therapy ball. Subsequently, they practiced multi-movement sequences, followed by integrative activities such as rolling while pushing a ball or rolling while striking a ball.  **Motor training:** children engaged in activities such as puzzles, object assembly, bead threading, and needle threading. Each session was scheduled for 30 minutes and conducted twice daily. For visual, auditory, and memory training, children practiced attention skills using cards containing text, figures, and numbers. | 1. Ensures physical safety. **YES**  2. Presents sensory opportunities. **YES**  3. Helps the child to attain and maintain appropriate levels of alertness. **YES**  4. Challenges postural, ocular, oral, or bilateral motor control. **YES**  5. Challenges in praxis and organization of behavior **No**  6. Collaborates in activity choice **Not clear**  7. Tailors activity to present just-right challenge **YES**  8. Ensures that activities are successful **YES**  9. Supports child’s intrinsic motivation to play. **YES**  10. Establishes a therapeutic alliance **YES** |
| Li Xiaoyan,2018 | **Not reported** | **Proprioceptive function training:** With the assistance of medical staff, children were guided to perform balanced exercises, such as walking on a balance beam. These activities were designed to improve balance, promote the maturation of the vestibular nervous system, and facilitate coordination with other neural systems. Indirectly, these exercises also supported the development of auditory and visual neural pathways.  **Tactile function training:** Children were guided to play with tactile boards, massage balls, and textured materials to stimulate the development of cutaneous sensory neural pathways, strengthen tactile feedback from muscles and joints, and enhance proprioceptive awareness. These exercises are further aimed to improve fine tactile discrimination and sensory refinement.  **Comprehensive training** Children were guided to participate in integrated activities, such as ball games, climbing, jumping, and other exercises. These tasks were intended to enhance overall motor coordination, improve jumping ability, regulate proprioceptive neural pathways, and facilitate the integration of muscle groups involved in motor control. Such activities promote the integration of sensory information within the brain, support the development of the central and peripheral nervous systems, and enhance the regulatory capacity of the neuromuscular system.  **Social and language function training:** Training sessions incorporated playful scenarios to enhance interest and engagement. During the sessions, children engaged in simple dialogues, and their verbal expressions were corrected as needed. Positive reinforcement and encouragement were provided to children who demonstrated good performance. | 1. Ensures physical safety. **YES**  2. Presents sensory opportunities. **YES**  3. Helps the child to attain and maintain appropriate levels of alertness. **NOT Clear**  4. Challenges postural, ocular, oral, or bilateral motor control. **YES**  5. Challenges in praxis and organization of behavior **NO**  6. Collaborates in activity choice **NOT Clear**  7. Tailors activity to present just-right challenge **YES**  8. Ensures that activities are successful **YES**  9. Supports child’s intrinsic motivation to play. **YES**  10. Establishes a therapeutic alliance **YES** |
| Zhang Yanmin,2018 | **Not reported** | **Tactile function training:** Large-area skin stimulation was applied to children with autism using tactile balls, ball pools, and similar activities, with the aim of improving tactile function.  **Vestibular function training:** Children were guided to perform rotational exercises. In a standing position, they were instructed to rotate the whole body or specific body parts around a relatively fixed axis to the left or right while keeping the feet stationary. Additional activities included standing on suspended equipment such as swinging bridges or barrels to practice rotation, rolling on the ground in a supine position with simultaneous foot movements, trampoline jumping, prone vibration on a large therapy ball, and bouncing on a therapy ball to provide up-and-down and vibratory stimulation.  **Proprioceptive function training:** Initially, children were instructed to repeat single-movement exercises, such as prone sliding on a scooter board or back rolling on a large therapy ball. Subsequently, they practiced multi-movement sequences, followed by integrative activities such as rolling while pushing a ball or rolling while striking a ball.  **Social and language function training:** Training sessions incorporated playful scenarios to enhance interest and engagement. During the sessions, children engaged in simple dialogues, and their verbal expressions were corrected as needed. Positive reinforcement and encouragement were provided to children who demonstrated good performance. | 1. Ensures physical safety. **YES**  2. Presents sensory opportunities. **YES**  3. Helps the child to attain and maintain appropriate levels of alertness. **YES**  4. Challenges postural, ocular, oral, or bilateral motor control. **YES**  5. Challenges in praxis and organization of behavior **NO**  6. Collaborates in activity choice **NO**  7. Tailors activity to present just-right challenge **YES**  8. Ensures that activities are successful **YES**  9. Supports child’s intrinsic motivation to play. **YES**  10. Establishes a therapeutic alliance **YES** |
| He Fengying,2019 | Yes | **Tactile function training:** Children are guided to use tactile balls, ball pits, and similar equipment to provide large-area stimulation to the skin surface, thereby improving tactile function.  **Proprioceptive function training:** Children are instructed to perform repetitive single-movement exercises, such as back rolling on a large therapy ball or prone sliding on a scooter board. They are then encouraged to engage in multi-movement sequences and integrative tasks, such as rolling while striking a ball or rolling while pushing a ball.  **Vestibular function training:** Children are guided to perform rotation exercises in a standing position, keeping their feet stationary while turning the body left or right. They may also practice rotations while standing on unstable equipment such as balance boards or barrels, or while lying on the ground rolling and turning with simultaneous foot movements. Additional activities may include assisted vibration and bouncing exercises on a therapy ball.  **Social and language function training:** Therapists incorporate playful and engaging activities into social and language exercises to attract the attention of children and stimulate their interest. Therapists are encouraged to interact frequently with children, engage in dialogue, correct inaccurate language use, and provide timely encouragement and praise. These strategies are intended to enhance confidence and motivation in children during sensory integration training. | 1. Ensures physical safety. **YES**  2. Presents sensory opportunities. **YES**  3. Helps the child to attain and maintain appropriate levels of alertness. **YES**  4. Challenges postural, ocular, oral, or bilateral motor control. **YES**  5. Challenges in praxis and organization of behavior **NO**  6. Collaborates in activity choice **NO**  7. Tailors activity to present just-right challenge **YES**  8. Ensures that activities are successful **YES**  9. Supports child’s intrinsic motivation to play. **YES**  10. Establishes a therapeutic alliance **YES** |
| Huang Yimin,2019 | **Not reported** | **Tactile function training:** Large-area skin stimulation was applied to children with autism using tactile balls, ball pools, and similar activities, with the aim of improving tactile function.  **Vestibular function training:** Children were guided to perform rotational exercises. In a standing position, they were instructed to rotate the whole body or specific body parts around a relatively fixed axis to the left or right while keeping the feet stationary. Additional activities included standing on suspended equipment such as swinging bridges or barrels to practice rotation, rolling on the ground in a supine position with simultaneous foot movements, trampoline jumping, prone vibration on a large therapy ball, and bouncing on a therapy ball to provide up-and-down and vibratory stimulation.  **Proprioceptive function training:** Initially, children were instructed to repeat single-movement exercises, such as prone sliding on a scooter board or back rolling on a large therapy ball. Subsequently, they practiced multi-movement sequences, followed by integrative activities such as rolling while pushing a ball or rolling while striking a ball.  **Social and language function training:** Training sessions incorporated playful scenarios to enhance interest and engagement. During the sessions, children engaged in simple dialogues, and their verbal expressions were corrected as needed. Positive reinforcement and encouragement were provided to children who demonstrated good performance. | 1. Ensures physical safety. **YES**  2. Presents sensory opportunities. **YES**  3. Helps the child to attain and maintain appropriate levels of alertness. **YES**  4. Challenges postural, ocular, oral, or bilateral motor control. **YES**  5. Challenges in praxis and organization of behavior **NOT Clear**  6. Collaborates in activity choice **NO**  7. Tailors activity to present just-right challenge **YES**  8. Ensures that activities are successful **YES**  9. Supports child’s intrinsic motivation to play. **YES**  10. Establishes a therapeutic alliance **YES** |
| Li Haiyu,2019 | **Not reported** | Sensory integration training was employed, with exercises including tactile, trampoline, and vestibular–balance activities. **Tactile training:** Children were guided to use tactile boards and massage balls, which help improve tactile function, promote the development of somatosensory neural pathways in the cortex, and enhance tactile sensitivity. **Vestibular balance training:** Children were instructed to perform activities such as standing on a balance beam, walking, and related tasks to improve balance, facilitate the maturation of the vestibular nervous system, and promote coordination of the central nervous system. **Trampoline training:** Children were guided to perform trampoline exercises, including jumping and bouncing, to improve jumping ability, regulate proprioceptive neural pathways, and strengthen coordination between muscle groups. These activities also enhance the integration of sensory information in the cerebrum and cerebellum, promoting the development of neural pathways within the central and cerebellar nervous systems.  **Proprioceptive function training:** Children are instructed to perform repetitive single-movement exercises, such as back rolling on a large therapy ball or prone sliding on a scooter board. They are then encouraged to engage in multi-movement sequences and integrative tasks, such as rolling while striking a ball or rolling while pushing a ball. | 1. Ensures physical safety. **YES**  2. Presents sensory opportunities. **YES**  3. Helps the child to attain and maintain appropriate levels of alertness. **YES**  4. Challenges postural, ocular, oral, or bilateral motor control. **YES**  5. Challenges in praxis and organization of behavior **NO**  6. Collaborates in activity choice **NO**  7. Tailors activity to present just-right challenge **YES**  8. Ensures that activities are successful **YES**  9. Supports child’s intrinsic motivation to play. **YES**  10. Establishes a therapeutic alliance **YES** |
| Li Huihui,2021 | **Not reported** | **Tactile function training:** Children are guided to use tactile balls, ball pits, and similar equipment to provide large-area stimulation to the skin surface, thereby improving tactile function.  **Proprioceptive function training:** Children are instructed to perform repetitive single-movement exercises, such as back rolling on a large therapy ball or prone sliding on a scooter board. They are then encouraged to engage in multi-movement sequences and integrative tasks, such as rolling while striking a ball or rolling while pushing a ball.  **Vestibular function training:** Children are guided to perform rotation exercises in a standing position, keeping their feet stationary while turning the body left or right. They may also practice rotations while standing on unstable equipment such as balance boards or barrels, or while lying on the ground rolling and turning with simultaneous foot movements. Additional activities may include assisted vibration and bouncing exercises on a therapy ball.  **Social and language function training:** Therapists incorporate playful and engaging activities into social and language exercises to attract the attention of children and stimulate their interest. Therapists are encouraged to interact frequently with children, engage in dialogue, correct inaccurate language use, and provide timely encouragement and praise. These strategies are intended to enhance confidence and motivation in children during sensory integration training. | 1. Ensures physical safety. **YES**  2. Presents sensory opportunities. **YES**  3. Helps the child to attain and maintain appropriate levels of alertness. **YES**  4. Challenges postural, ocular, oral, or bilateral motor control. **YES**  5. Challenges in praxis and organization of behavior **Not clear**  6. Collaborates in activity choices **NO**  7. Tailors activity to present just-right challenge **YES**  8. Ensures that activities are successful **YES**  9. Supports child’s intrinsic motivation to play. **YES**  10. Establishes a therapeutic alliance **YES** |
| Wan Kai,2021 | **Not reported** | **Tactile training:** Tactile defensiveness training: Includes walking, running, jumping, and crawling combined with tactile equipment. Examples: therapy brushing, tactile ball standing, tactile board crawling, jumping, sandbags, soft objects, and ocean ball pools.  Tactile under-responsivity training: Activities such as tactile ball standing, tactile board crawling, jumping, sandbags, soft materials, and ocean ball pools.  **Vestibular sensation training:** Balance ability training: Includes walking, running, jumping, and crawling. Examples: single-leg stance, double-leg jumps, hurdle crossing, etc.  **Proprioception training:** Jumping ability training: Examples: goat vaulting, trampoline jumping, balance platform, balance beam, and dynamic balance rockers.  Motor control training: Examples: balance beam, indoor climbing wall, swing sets, climbing frame combinations, rope ladders, toy car walking, spiral climbing, slide tunnels, and unicycle chairs.  Coordination training: Emphasizes multi-movement tasks combining balance, locomotion, and coordination.  **Language training:** This includes gaze training, articulation and speech organ exercises, naming tasks, and family participation.  SII includes the specific training content mentioned above, with a focus on the same internal therapeutic mechanism and multiple different games as carriers. The specific instruments used can have various | 1. Ensures physical safety. **YES**  2. Presents sensory opportunities. **YES**  3. Helps the child to attain and maintain appropriate levels of alertness. **YES**  4. Challenges postural, ocular, oral, or bilateral motor control. **YES**  5. Challenges in praxis and organization of behavior **NO**  6. Collaborates in activity choice **NO**  7. Tailors activity to present just-right challenge **YES**  8. Ensures that activities are successful **YES**  9. Supports child’s intrinsic motivation to play. **YES**  10. Establishes a therapeutic alliance **YES** |
| Zhang Xiaoyu,2022 | **Not reported** | **Tactile training:** Tactile defensiveness training: Includes walking, running, jumping, and crawling combined with tactile equipment. Examples: therapy brushing, tactile ball standing, tactile board crawling, jumping, sandbags, soft objects, and ocean ball pools.  Tactile under-responsivity training: Activities such as tactile ball standing, tactile board crawling, jumping, sandbags, soft materials, and ocean ball pools.  **Vestibular sensation training:** Balance ability training: Includes walking, running, jumping, and crawling. Examples: single-leg stance, double-leg jumps, hurdle crossing, etc.  **Proprioception training:** Jumping ability training: Examples: goat vaulting, trampoline jumping, balance platform, balance beam, and dynamic balance rockers.  Motor control training: Examples: balance beam, indoor climbing wall, swing sets, climbing frame combinations, rope ladders, toy car walking, spiral climbing, slide tunnels, and unicycle chairs.  Coordination training: Emphasizes multi-movement tasks combining balance, locomotion, and coordination.  **Vision training:** Hand-eye coordination training: Paired with motor training, often integrated with vestibular and proprioceptive exercises. Examples: ball catching, color tracking, figure discrimination, and visual memory tasks.  Visual analysis training: Includes color recognition, figure–ground discrimination, and visual memory.  **Hearing training:** Auditory training: Includes listening to music, distinguishing sounds, perceiving natural environmental sounds, and auditory memory tasks.  **Language training:** This includes gaze training, articulation and speech organ exercises, naming tasks, and family participation. | 1. Ensures physical safety. **YES**  2. Presents sensory opportunities. **YES**  3. Helps the child to attain and maintain appropriate levels of alertness. **NOT Clear**  4. Challenges postural, ocular, oral, or bilateral motor control. **YES**  5. Challenges in praxis and organization of behavior **NO**  6. Collaborates in activity choice **NOT Clear**  7. Tailors activity to present just-right challenge **YES**  8. Ensures that activities are successful **YES**  9. Supports child’s intrinsic motivation to play. **YES**  10. Establishes a therapeutic alliance **YES** |
| Zhang Guixin,2020 | **Not reported** | **Sensory training:** Through the use of equipment such as slides, balance beams, peg-insertion boards, trampolines, unicycle chairs, jump ropes, and sensory massage exercises, the child’s vestibular, auditory, visual, and proprioceptive systems are targeted. In parallel, multimedia technology is employed: videos and music are used to prompt and guide dance movements. Additionally, games that train limb coordination—such as bead threading, assembly tasks, puzzles, and sewing-through-hoop tasks.  **Proprioceptive function training:** Children are instructed to perform repetitive single-movement exercises, such as back rolling on a large therapy ball or prone sliding on a scooter board. They are then encouraged to engage in multi-movement sequences and integrative tasks, such as rolling while striking a ball or rolling while pushing a ball.  **Vestibular function training:** Children are guided to perform rotation exercises in a standing position, keeping their feet stationary while turning the body left or right. They may also practice rotations while standing on unstable equipment such as balance boards or barrels, or while lying on the ground rolling and turning with simultaneous foot movements. Additional activities may include assisted vibration and bouncing exercises on a therapy ball.  **Language training:** This includes gaze training, articulation and speech organ exercises, naming tasks, and family participation.  **Gaze training:** Using toys, food, or other preferred items to attract the child’s visual attention and guide them to approach the trainer.  **Articulation / speech organ training:** Includes instructing the child to open and close the mouth, protrude and retract the tongue, curl the tongue, and tapping facial muscles or lips. The child is further guided to imitate, read aloud, or repeat language, transitioning from single words to sentences and then to paragraphs.  **Naming tasks:** Multiple images are placed before the child. According to verbal instructions, the child identifies the correct image, progressing from two-choice to four-choice, to six-choice, and eventually to ten-choice tasks. The images are chosen to reflect everyday life to encourage meaningful communication.  **Family involvement:** Parents are guided to engage in regular communication with the child, applying the knowledge and skills learned in training to everyday life. | 1. Ensures physical safety. **YES**  2. Presents sensory opportunities. **YES**  3. Helps the child to attain and maintain appropriate levels of alertness. **YES**  4. Challenges postural, ocular, oral, or bilateral motor control. **YES**  5. Challenges in praxis and organization of behavior **NO**  6. Collaborates in activity choice **NO**  7. Tailors activity to present just-right challenge **YES**  8. Ensures that activities are successful **YES**  9. Supports child’s intrinsic motivation to play. **YES**  10. Establishes a therapeutic alliance **YES** |
| Pi Xiang,2020 | **Not reported** | **Tactile training:** Tactile defensiveness training: Includes walking, running, jumping, and crawling combined with tactile equipment. Examples: therapy brushing, tactile ball standing, tactile board crawling, jumping, sandbags, soft objects, and ocean ball pools.  Tactile under-responsivity training: Activities such as tactile ball standing, tactile board crawling, jumping, sandbags, soft materials, and ocean ball pools.  **Vestibular sensation training:** Balance ability training: Includes walking, running, jumping, and crawling. Examples: single-leg stance, double-leg jumps, hurdle crossing, etc.  **Proprioception training:** Jumping ability training: Examples: goat vaulting, trampoline jumping, balance platform, balance beam, and dynamic balance rockers.  Motor control training: Examples: balance beam, indoor climbing wall, swing sets, climbing frame combinations, rope ladders, toy car walking, spiral climbing, slide tunnels, and unicycle chairs.  Coordination training: Emphasizes multi-movement tasks combining balance, locomotion, and coordination.  **Vision training:** Hand-eye coordination training: Paired with motor training, often integrated with vestibular and proprioceptive exercises. Examples: ball catching, color tracking, figure discrimination, and visual memory tasks.  Visual analysis training: Includes color recognition, figure–ground discrimination, and visual memory.  **Hearing training:** Auditory training: Includes listening to music, distinguishing sounds, perceiving natural environmental sounds, and auditory memory tasks.  SII includes the specific training content mentioned above, with a focus on the same internal therapeutic mechanism and multiple different games as carriers. The specific instruments used can have various combinations, and the use of instruments can be adjusted in a timely and personalized manner according to children's reactions. | 1. Ensures physical safety. **YES**  2. Presents sensory opportunities. **YES**  3. Helps the child to attain and maintain appropriate levels of alertness. **YES**  4. Challenges postural, ocular, oral, or bilateral motor control. **YES**  5. Challenges in praxis and organization of behavior **NO**  6. Collaborates in activity choice **NO**  7. Tailors activity to present just-right challenge **YES**  8. Ensures that activities are successful **YES**  9. Supports child’s intrinsic motivation to play. **YES**  10. Establishes a therapeutic alliance **YES** |
| Wenxin Xu,2019 | **Not reported** | According to the clinical characteristics of autism, this method is mainly embodied in exercise games. It uses games such as slides, tossing, leaping, balance beam, and sling to train children’s balance, communication, and brain integration functions. While exercising, objects are required to assist each other in completing various exercise programs, thereby promoting their communication. Suitable exercise programs are selected in accordance with the conditions and hobbies of children with autism to attract their attention and promote the gradual recovery of their communication skills. Given the large number of members in the group exercise programs, more communication would be required. Patients can be guided to participate in group exercise programs to increase their mutual familiarity and to achieve therapeutic goals at the same time. These exercise programs do not only require patients to participate actively but also try to get the support of their parents. In doing so, parents can observe the gradual changes exhibited by patients. Thus, parents can adjust accordingly and help in promoting the recovery of the patients. | 1. Ensures physical safety. **YES**  2. Presents sensory opportunities. **YES**  3. Helps the child to attain and maintain appropriate levels of alertness. **YES**  4. Challenges postural, ocular, oral, or bilateral motor control. **YES**  5. Challenges in praxis and organization of behavior **YES**  6. Collaborates in activity choice **NO**  7. Tailors activity to present just-right challenge **YES**  8. Ensures that activities are successful **NOT Clear**  9. Supports child’s intrinsic motivation to play. **YES**  10. Establishes a therapeutic alliance **YES** |
